# Volume Holograms in Photopolymers: Comparison between Analytical and Rigorous Theories

**DOI:** 10.3390/ma5081373

**Published:** 2012-08-15

**Authors:** Sergi Gallego, Cristian Neipp, Luis A. Estepa, Manuel Ortuño, Andrés Márquez, Jorge Francés, Inmaculada Pascual, Augusto Beléndez

**Affiliations:** 1 Department of Physics, Systems Engineering and Signal Theory, University of Alicante, Apartado 99, E03080 Alicante, Spain; E-Mails: cristian@dfists.ua.es (C.N.); luisestepa@gmail.com (L.A.E.); mos@ua.es (M.O.); andres.marquez@ua.es (A.M.); jfmonllor@ua.es (J.F.); a.belendez@ua.es (A.B.); 2 University Institute of Physics Applied to Sciences and Technologies, University of Alicante, Apartado 99, E03080 Alicante, Spain; 3 Department of Optics, Pharmacology and Anatomy, University of Alicante, Apartado 99, E03080 Alicante, Spain; E-Mail: pascual@ua.es

**Keywords:** holographic materials, photopolymers, volume holograms

## Abstract

There is no doubt that the concept of volume holography has led to an incredibly great amount of scientific research and technological applications. One of these applications is the use of volume holograms as optical memories, and in particular, the use of a photosensitive medium like a photopolymeric material to record information in all its volume. In this work we analyze the applicability of Kogelnik’s Coupled Wave theory to the study of volume holograms recorded in photopolymers. Some of the theoretical models in the literature describing the mechanism of hologram formation in photopolymer materials use Kogelnik’s theory to analyze the gratings recorded in photopolymeric materials. If Kogelnik’s theory cannot be applied is necessary to use a more general Coupled Wave theory (CW) or the Rigorous Coupled Wave theory (RCW). The RCW does not incorporate any approximation and thus, since it is rigorous, permits judging the accurateness of the approximations included in Kogelnik’s and CW theories. In this article, a comparison between the predictions of the three theories for phase transmission diffraction gratings is carried out. We have demonstrated the agreement in the prediction of CW and RCW and the validity of Kogelnik’s theory only for gratings with spatial frequencies higher than 500 lines/mm for the usual values of the refractive index modulations obtained in photopolymers.

## 1. Introduction

Photopolymers are useful for different applications due to the refractive index variations and relief profiles generated [1,2,3,4,5,6,7,8]. There are many types of photopolymers that may be differentiated by the type of binder, since this component determines to a great extent the choice of monomer, dye and initiator used in the photopolymer. Normally, these materials are used in holographic applications, where high values of spatial frequencies are recorded. The characterization of the parameters that govern the hologram formation in the layer has been classically done recording volume phase gratings [9,10]. The main advantage of this method is that characterization and optimization of the material, and of the processes to store recorded holographic gratings, are performed simultaneously. For example, it is possible to multiplex many gratings in the material, trying to achieve high values of diffraction efficiency, and to measure the signal-to-noise ratio. Using this method to characterize photopolymers as holographic materials the fittings of the gratings stored in them are of great importance. Using these fittings, parameters such as the refractive index modulation, optical thickness and the scattering coefficients can be calculated. Thus discrepancies between physical and optical thickness can be analyzed [11] and also the multiplexing methods for holographic data storage can be improved [12].

In addition, many spatial frequencies and refractive index modulations can be recorded in these materials [13,14,15,16,17]. With these studies the utility of photopolymers for a wide range of applications has been demonstrated, from diffractive optical elements in the very low spatial frequencies range to reflection gratings in the high spatial frequency range. Therefore we need a useful theory to explain the propagation of the light inside the grating, to accurately fit all of the hologram parameters. There are many theories used to achieve this goal, but some times the limits of the applicability for each method to analyze the gratings recorded in photopolymers are not well defined. For example historically, the Kogelnik’s Coupled Wave theory [18] has been used to analyze the gratings recorded in photopolymers [19,20]. Nevertheless, since its formulation, a more rigorous theory, the Rigorous Coupled Wave theory (RCW) [21], has also been applied to analyze these gratings [22,23]. In the first work [22] the authors have demonstrated that the higher harmonics in the refractive index modulation can be neglected, and in the second work [23] the authors have shown that the errors can even be higher than 30% if Kogelnik’s theory is used in the characterization of the gratings.

Kogelnik’s Coupled Wave theory has the advantage over other theories that, in spite of being mathematically simple, it predicts very accurately the response of the efficiency of the zero and first orders for volume phase gratings. Nonetheless, the accuracy decreases when either the thickness is low or when over-modulated patterns (high refractive index modulations) are recorded in the hologram. It is therefore necessary to study the limits of applicability of Kogelnik’s theory to the study of gratings recorded in photopolymers.

To establish a criterion for the applicability of Kogelnik’s theory two parameters should be compared, the volume factor, *Q*, and the grating strength, *v* [24,25,26]. Two different curves in the *Q*–*ν*, plane, *νQ* = const, *Q/ν* = const, delimit the Raman-Nath and Bragg regimes. In the Bragg regime Kogelnik’s theory is highly applicable.

In this work we will study the applicability of Kogelnik’s Coupled Wave theory to gratings recorded in photopolymers for usual values of parameters such as thickness or refractive index modulation. The curves obtained by using Kogelnik’s theory will be compared to those obtained by using the RCW theory and the CW theory (the second derivatives are disregard) [27,28,29]. In addition, experimental data of the angular responses of the first and zero orders for diffraction gratings recorded in photopolymers are fitted by using Kogelnik’s and RCW theories. To carry out the experimental work we have used a polyvinyl-alcohol/acrylamide (PVA/AA) based photopolymer.

## 2. Theoretical Background

In our theoretical analysis we assume that only the first harmonic of the electric permittivity is significant and that the zone of analysis is near to the first Bragg’s angle. Then the relative electric permittivity can be written as:

(1) $$\varepsilon_{r}(r)=\varepsilon_{r0}+\varepsilon_{r1}\cos(K\cdot r)$$

where ***K*** is the wave vector and can be expressed as function of the grating period; *Λ*, or as function of the frequency, *f*:

(2) $$\left|K\right|=\frac{2\pi }{\Lambda }=2\pi f$$

In the case of volume gratings the two coupled wave Kogelnik’s theory allows us finding analytical expressions of the diffraction efficiency as function of the angle [18]. The diffraction efficiency, *η*, of the first order, in the case of non-slanted phase gratings is given by:

(3) $$\eta =\exp\left(\frac{-\alpha d}{\cos\theta }\right)\frac{\sin^{2}\sqrt{\nu^{2}+\xi^{2}}}{1+(\xi^{2}/\nu^{2})}$$

where *d* is the thickness, *θ* the incidence angle inside the medium, and *ν*, the grating strength, and *ξ* are given by:

(4) $$\nu =\frac{\;\pi \;d\varepsilon_{r1}}{2\lambda_{0}\;\varepsilon_{r0}^{1/2}\cos\theta }=\frac{\pi \;n_{1}d}{\lambda_{0}\cos\theta }$$

(5) $$\xi =\frac{\vartheta \;d}{2\cos\theta }$$

In these equations *n*_1_ is the refractive index modulation; *λ*_0_ is the wavelength in vacuum; and *ϑ* is a parameter that measures the Bragg angle deviation.

When other orders are considered in a more accurate analysis, we assume that the electric field can be written as the sum of the contributions of the different orders:

(6) $$E_{1}=\sum_{i}S_{i}(z)\exp(-j\rho_{i}\cdot r)\;i=0,\pm 1,\pm 2,...$$

and the propagation vectors of each order can be obtained using Floquet condition:

***ρ**_i_* = ***ρ***_0_+*i**K***    *i* = 0, ±1, ±2,... (7)

It is important to notice that Equation (7) can only be applied, to beaccurate, in the case of reconstruction at Bragg angle when using Kogelnik’s Coupled Wave Theory and the Coupled Wave Theory. When a plane wave impinges onthe grating out of Bragg angle Equation (7) would lead to incorrect predictions (inelastic diffraction), for a better explanation on this issue see [30].

The equation that governs the behavior of the several diffracted orders propagating inside the periodic medium can be expressed as [27,31,32]:

(8) $$\frac{j}{2\beta }\frac{d^{2}S_{i}(z)}{dz^{2}}+C_{i}\frac{dS_{i}(z)}{dz}-j\kappa \Omega i(i+P)S_{i}(z)+j\kappa (S_{i+1}(z)+S_{i-1}(z))=0$$

where *C*_i_ are the called obliquity factors and are the cosine of the angles that the propagation vectors of the different orders form with the *z*-axis. In the particular case of non-slanted diffraction gratings, *C*_i_ = cos*θ*_0_, where *θ*_0_ is the angle of reconstruction inside the medium.

The parameter *κ* is the coupling constant and is defined as:

(9) $$\kappa =\frac{\beta \varepsilon_{r1}}{4\varepsilon_{r0}}$$

Here *β* is the propagation constant inside the grating.

The *Ω* parameter is defined as:

(10) $$\Omega =\frac{{\left|K\right|}^{2}}{2\beta \kappa }$$

As has been demonstrated by Solymar and Cooke [27], *Ω* is a parameter, which gives a criterion of whether the hologram is thin or thick. It is stated in reference [27] that whenever *Ω* > 5 the hologram can be considered thick. Finally, the parameter *P* is defined as:

(11) $$P=\frac{2\beta }{\left|K\right|}\text{sin}(\theta_{0}-\varphi )$$

where *φ* is the angle between the fringes and the z-axis, which for non-slanted geometry is 0; the parameter *P* is the called impact parameter and takes the values; *P* = 1 for reconstruction at first Bragg angle; *P* = 2 at the second Bragg angle, and so on. In this work we shall restrict the study to the first Bragg condition.

It as been a common strategy in volume holography to disregard the second derivatives of Equation (8), since slow variation of the diffracted orders inside the grating is supposed. This is not completely true for high values of the coupling constant, *κ*, but as will be demonstrated in this study in the range of values that will be treated here, this approximation is perfectly valid.

If the second derivatives are disregarded Equation (8) transforms into:

(12) $$\frac{dS_{i}(z)}{d\zeta }-j\Omega i(i+P)S_{i}(\zeta )+j(S_{i+1}(\zeta )+S_{i-1}(\zeta ))=0$$

where:

(13) $$\zeta =\frac{\kappa z}{\cos\theta_{0}}$$

Now, Equation (12) has only two significant parameters, *Ω* and *P*. The influence of *Ω* and *P* can easily be interpreted from Equation (12). Coupling from one order to the two adjacent ones takes place through the last term of this equation. The importance of this term grows as *Ω* decreases, diminishing the influence of the second term. Therefore multi-wave diffraction occurs whenever *Ω* is small. Although this argument can be considered general, the rigorous Equation (8) cannot be expressed in terms of only two significant parameters, *Ω* and *P*, so a more complex interpretation must be made. Nonetheless, it can be seen that coupling from one order to the adjacent ones is strengthen whenever the value of *κ* increases, and the product *Ωκ* diminishes. Thus, for high values of *κ* and low values of *Q* multi-wave diffraction occurs.

On the other hand a rigorous solution of Equation (8) can be obtained by using the so-called Rigorous Coupled Wave theory (RCW). As has been demonstrated since its first introduction by Moharam and Gaylord [21] the RCW method has accomplished the task of explaining a great number of physical situations associated with diffraction gratings of different kind [33,34,35]. This theory is also useful to check into what extent the approximations made in other theories are valid. In its initial formulation the method proposed presented some stability problems when thick layers are considered. A stable formulation of the method was proposed by Moharam *et al.* in 1995 [35]. We have chosen this version of the Rigorous Coupled Wave theory (RCW) to simulate the propagation of the light in the gratings recorded in photopolymers.

It is interesting to take into account that the theories of CW and RCW assume a harmonic dependence of the relative dielectric permittivity with *x*, but in holography is usual to characterize the phase gratings using the refractive index. To compare accurately the three theories using the same parameters we can write the first harmonic of the relative dielectric permittivity as:

(14) $$\varepsilon_{1}=2n_{0}n_{1}$$

This approximation can be done when *n*_1_ << *n*_0_. The cases analyze in this work are consistent with condition.

## 3. Gratings Recorded in Photopolymers

### 3.1. Theoretical Simulations

In this section we will compare the solutions obtained by the three methods described, CW, RCW and Kogelnik’s theories applied to transmission gratings for different spatial frequencies (described in Table 1). The simulations were performed using parameters of refractive index modulation and thickness, usual in photopolymers. In the first case a value of the refractive index modulation *n*_1_ = 0.004 was used [36]. In the second place we performed the analysis with a value of the refractive index modulation *n*_1_ = 0.011, a value which is near of maximum for materials based in PVA/AA and is achieved using a crosslinker monomer, NN’,methylene-bis-acrylamide (BMA) [35,36].

**Figure 1:**
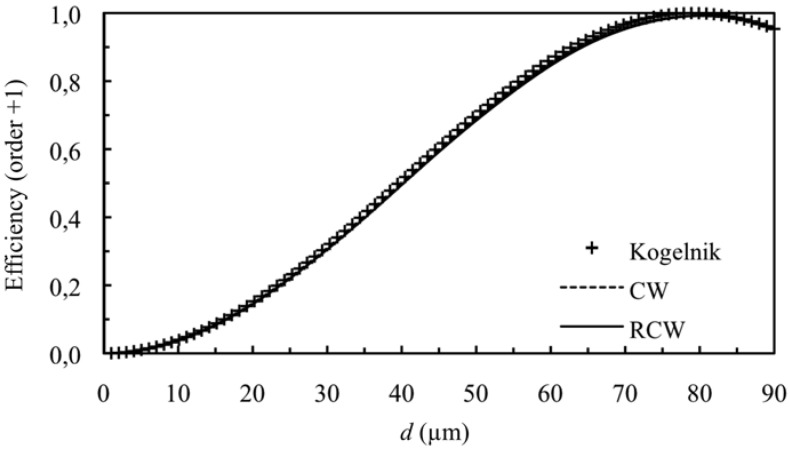
Diffraction efficiency of order +1 (first order diffracted), at first Bragg’s angle. The spatial frequency is 350 lines/mm and the refractive index modulation is *n*_1_ = 0.004.

**Figure 2:**
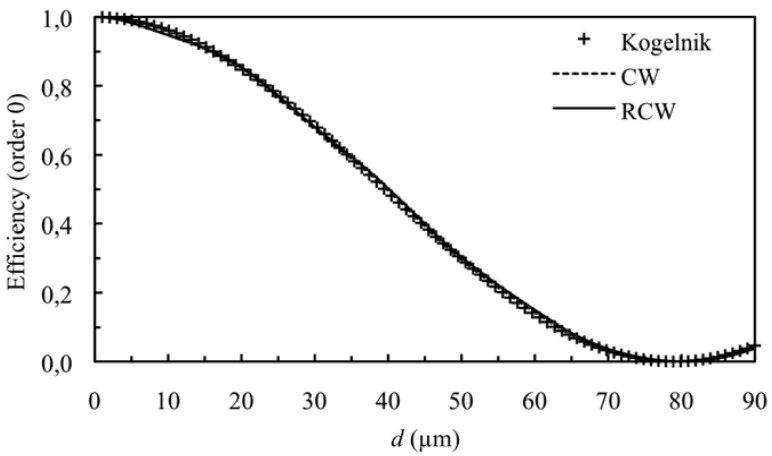
Efficiency of order 0 (zero order diffracted), at first Bragg’s angle. The spatial frequency is 350 lines/mm and the refractive index modulation is *n*_1_ = 0.004.

Figure 1 and Figure 2 show the diffraction efficiency as function of the thickness for the orders +1 and 0 for a spatial frequency of 350 lines/mm and a value of the refractive index modulation of *n*_1_ = 0.004, at first Bragg’s condition (*Ω* takes the value 8.3). The value of *Ω* shows that we are in Bragg’s regime, and as can be expected the predictions of the three theories are similar (Kogelnik, CW and RCW) in the range of thickness studied.

**Table 1:** Values of the parameters of the phase transmission gratings for different spatial frequencies.

Refractive index modulation	*f* = 350 lines/mm	*f* = 500 lines/mm	*f* = 750 lines/mm
*n*_1_ = 0.004	*Ω* ~ 8	*Ω* ~ 17	*Ω* ~ 38
*n*_1_ = 0.011	*Ω* ~ 3	*Ω* ~ 6	*Ω* ~ 14

**Figure 3:**
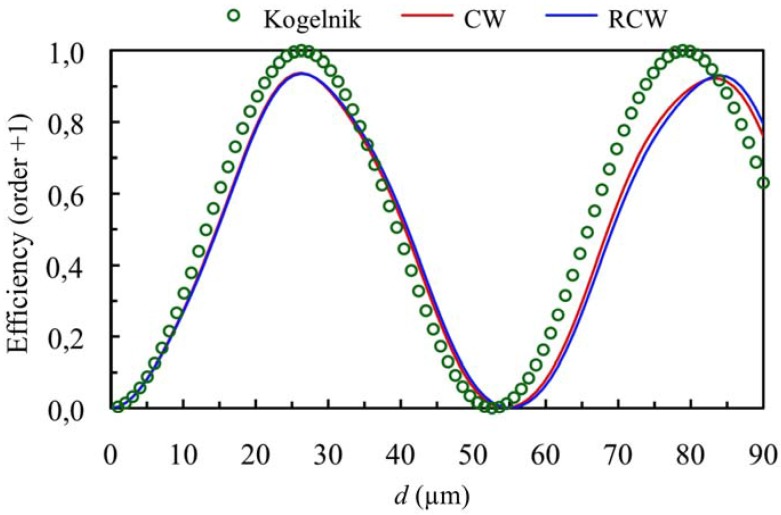
Diffraction efficiency of order +1 (first order diffracted), at first Bragg’s angle. The spatial frequency is 350 lines/mm and the refractive index modulation is *n*_1_ = 0.011.

**Figure 4:**
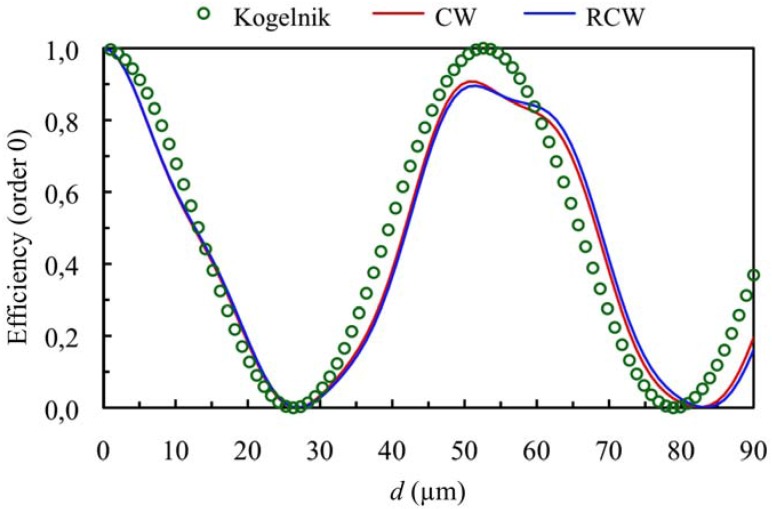
Efficiency of order 0 (zero order diffracted), at first Bragg’s angle. The spatial frequency is 350 lines/mm and the refractive index modulation is *n*_1_ = 0.011.

**Figure 5:**
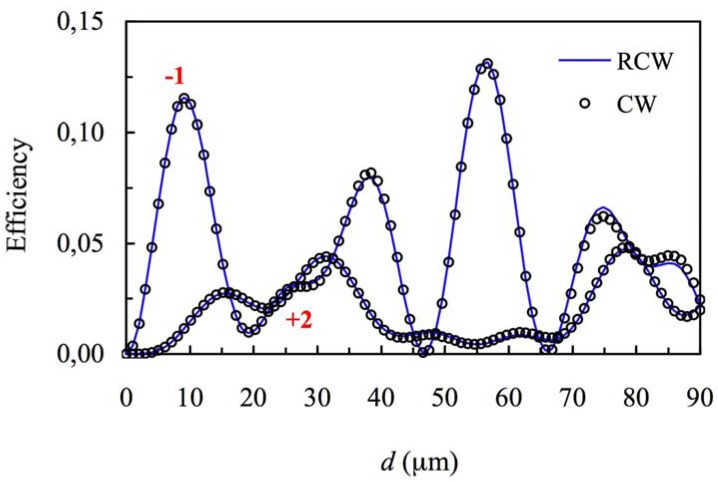
Efficiency of the orders −1 and +2, at first Bragg’s angle. The spatial frequency is 350 lines/mm and the refractive index modulation is *n*_1_ = 0.011.

When higher values of the refractive index modulation are considered (*n*_1_ = 0.011) for the same spatial frequency the curves presented in Figure 3 and Figure 4 can be obtained (first and zero orders *versus* thickness at first Bragg angle condition), *Ω* takes a value of 3, so this grating cannot be classified as a volume one. If these figures are analyzed we can see that there exist deviations between the predictions using Kogelnik’s theory and the results obtained using CW and RCW. This fact occurs because the secondary orders cannot be neglected and part of the energy is diffracted in other directions (secondary diffracted orders). It is also interesting to remark that in all the cases the predictions of CW and RCW are very similar, what shows that the second derivates can be neglected in Equation (8). The results obtained by these two theories are also similar for secondary orders. The energy of orders −1 and +2 as a function of the thickness of the grating are represented in Figure 5.

**Figure 6:**
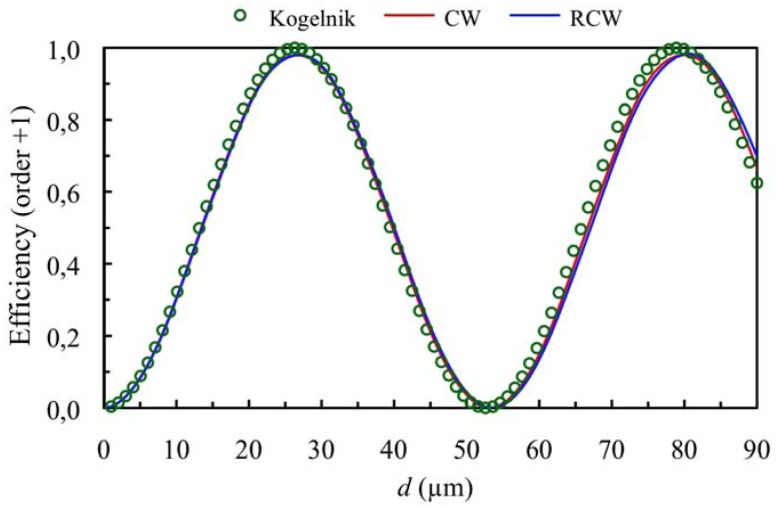
Diffraction efficiency of order +1 (first order diffracted), at first Bragg’s angle. The spatial frequency is 500 lines/mm and the refractive index modulation is *n*_1_ = 0.011.

**Figure 7:**
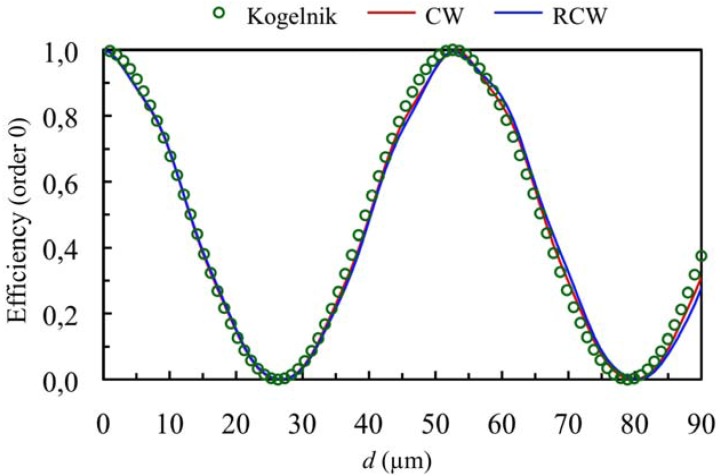
Efficiency of order 0 (zero order diffracted), at first Bragg’s angle. The spatial frequency is 500 lines/mm and the refractive index modulation is *n*_1_ = 0.011.

When higher values of the spatial frequency are considered (500 lines/mm) the values of the parameter *Ω* are higher than 5 (*Ω* = 6.2). The efficiencies of the orders +1 and 0 as a function of the thickness of the grating are represented in Figure 6 and Figure 7, respectively when high values of the index modulation are considered (*n*_1_ = 0.011). In these figures there is good agreement between the CW and RCW theories, but the results obtained by using Kogelnik Coupled Wave Theory slightly deviate from those of RCW and CW theories. This is due to the existence of multi-order diffraction, although the results are clearly closer than in the case of 350 lines/mm.

**Figure 8:**
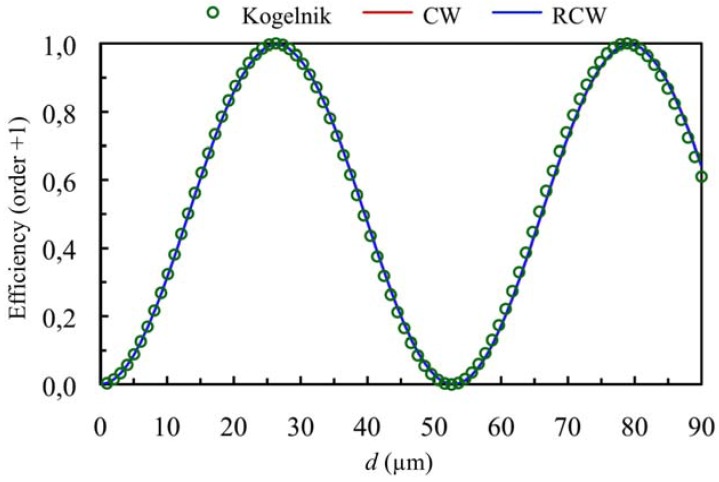
Diffraction efficiency of order +1 (first order diffracted), at first Bragg’s angle. The spatial frequency is 750 lines/mm and the refractive index modulation is *n*_1_ = 0.011.

**Figure 9:**
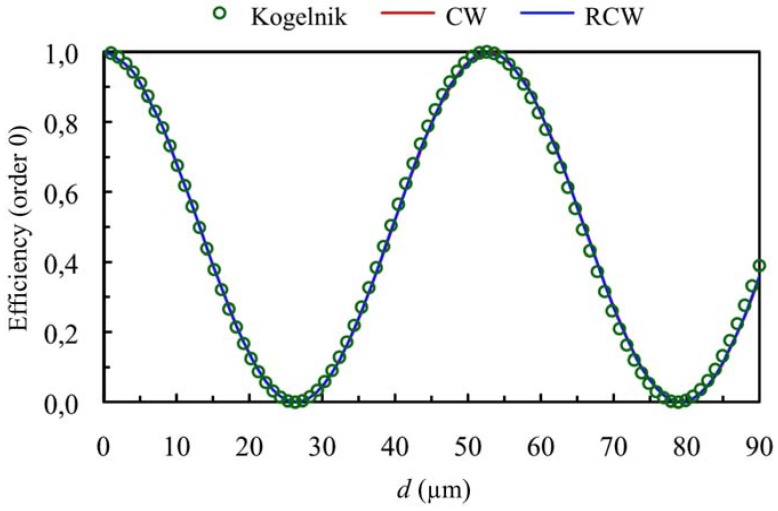
Efficiency of order 0 (zero order diffracted), at first Bragg’s angle. The spatial frequency is 750 lines/mm and the refractive index modulation is *n*_1_ = 0.011.

If the spatial frequencies considered are 750 lines/mm, the values of *Ω* are higher than 10. In Figure 8 and Figure 9 the orders +1 and 0 are represented when *n*_1_ = 0.011 (*Ω =* 14). It is evident that in this case the agreement between the three theories is almost perfect, so in this case Kogelnik’s theory is highly applicable to analyze these gratings at first Bragg’s angle.

### 3.2. Experimental Results

In this section we analyze the experimental results using RCW and Kogelnik’s theories, to check the validity of the information obtained using these theories. In our comparison with experimental data we analyzed the holographic behavior of an acrylamide photopolymer in layers that range in thickness from 20 to 90 μm and spatial frequency between 500 and 1125 lines/mm. The photopolymer is composed of acrylamide (AA) as polymerizable monomer, N,N’,methylene-bis-acrylamide (BMA) as crosslinker, triethanolamine (TEA) as radical generator, yellowish eosin (YE) as sensitizer and polyvinyl-alcohol (PVA) as binder. A solution of PVA in water forms the matrix and this is used to prepare the mixture of AA, BMA and photopolymerization initiator system (TEA, YE). The PVA was supplied by Fluka (Sigma-Aldrich Corporation, 3050 Spruce St., St. Louis, MO, USA), AA and TEA by Sigma (St. Louis, MO, USA) and YE by Panreac (Barcelona, Spain). The experimental procedure is described with more details in references [19,37].

The mixture is done under red light and after evaporation of part of the water; a solid plastic film is formed on a glass plate, which constitutes the holographic recording material the quantity of depositing the solution varies depending on the desired thickness of the final layer. For each thickness we analyzed the holographic behavior of the material during recording of non-slanted diffraction gratings using a continuous argon laser (514 nm) at an intensity of 5 mW/cm^2^.

**Figure 10:**
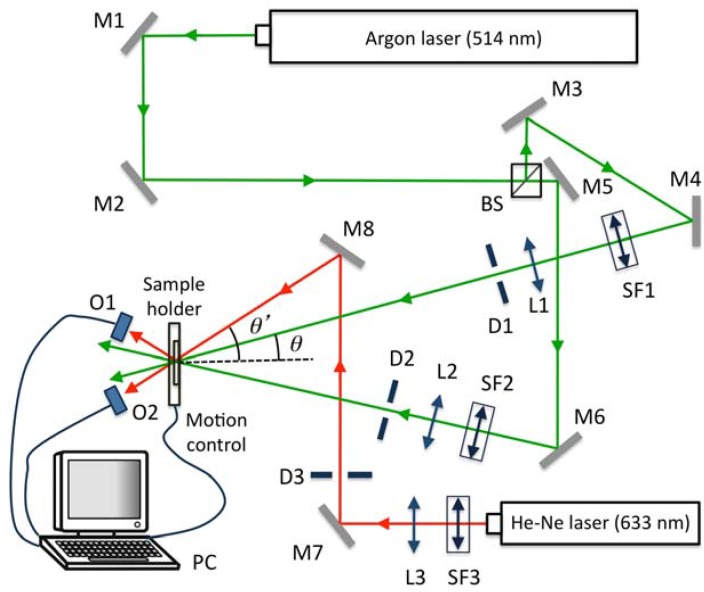
Experimental set-up for analyzing volume holograms in photopolymer.

**Figure 11:**
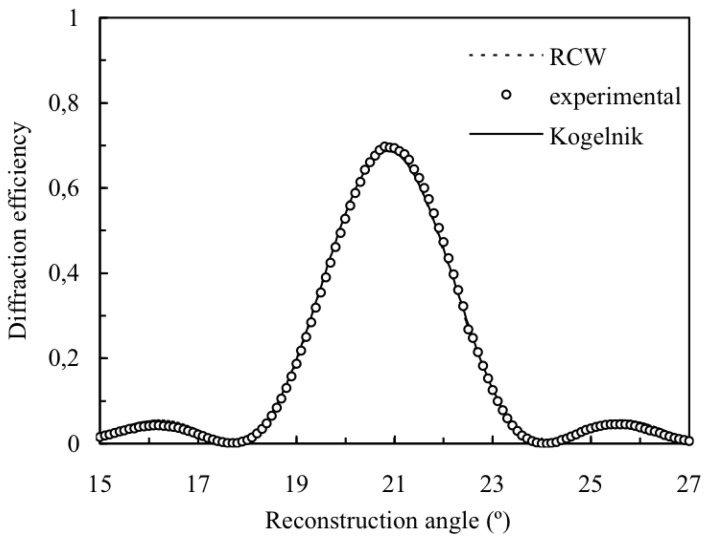
Diffraction efficiency of the order +1 for volume transmission gratings recorded in polyvinyl-alcohol/acrylamide (PVA/AA) with spatial frequency 1125 lines/mm and 23 µm of thickness.

The experimental device used is shown in Figure 10. The green beam was split into two secondary beams with an intensity ratio of 1:1. The diameters of these beams were increased to 1.5 cm with an expander, while spatial filtering was ensured. The angular response of each grating was monitored with a He-Ne laser. The angles between the object and reference beams and the red beam depend on the chosen spatial frequency. An usual value of the spatial frequency of a gratings recorded in a photopolymers material is around 1000 lines/mm, for this spatial frequency an index modulation higher than 0.01 can be obtained with low thickness (around 20 µm) when appropriated crosslinkers concentrations are used. The comparison between the experimental results for the angular response of the efficiency of order +1 and the theoretical values obtained using Kogelnik and RCW theories is presented in Figure 11 for a transmission diffraction grating with a spatial frequency of 1125 lines/mm. It is important to note that for off-Bragg replay Kogelnik’s Coupled Wave Theory presents some inconsistencies in its formulation (Equation 7 should not be applied in this case, see reference to a detail analysis of this fact [30]), nevertheless the deviations in the predicted diffraction efficiency are assumable. As can be seen, the results obtained are similar, and the parameters fitted are the same (*d* = 23 µm, *n*_1_ = 0.0102, and *α* = 0.0056 μm^−1^).

**Figure 12:**
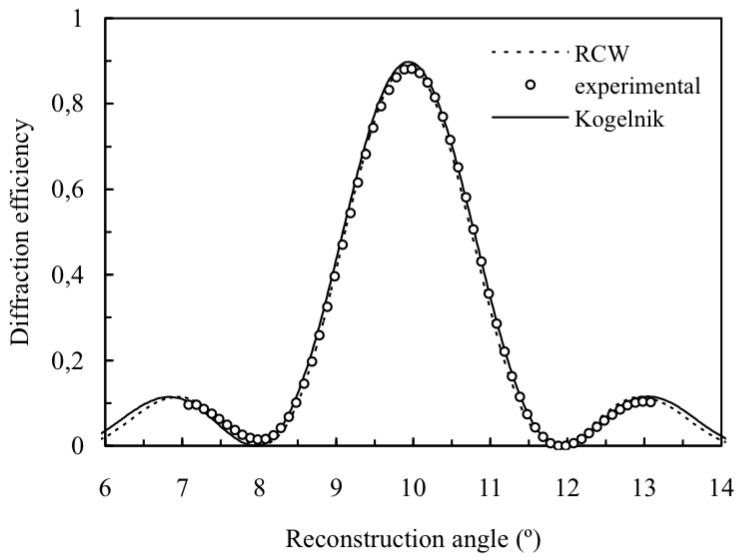
Diffraction efficiency of order +1 for volume transmission gratings recorded in PVA/AA with spatial frequency 545 lines/mm and 69 µm of thickness at first Bragg’s angle.

The existence of a low cut-off spatial frequency in photopolymers means that sometimes the theoretical values analyzed in the last section are difficult to obtain in photopolymers. In general it is difficult to work with spatial frequencies under 500 lines/mm with high values of the refractive index modulation because the diffusion is not an important process in this case, the index modulations are clearly lower than 0.01 [13,14]. Figure 12 shows the experimental diffraction efficiency of the order +1 around the first Bragg angle (in this case 9.88° when the wavelength used is 633 nm), the Kogelnik’s theory predictions and the RCW analysis. The values obtained by the two fittings are the same: *n*_1_ = 0.00476, *d* = 69 µm and *α* = 0.0042 μm^−1^.

**Figure 13:**
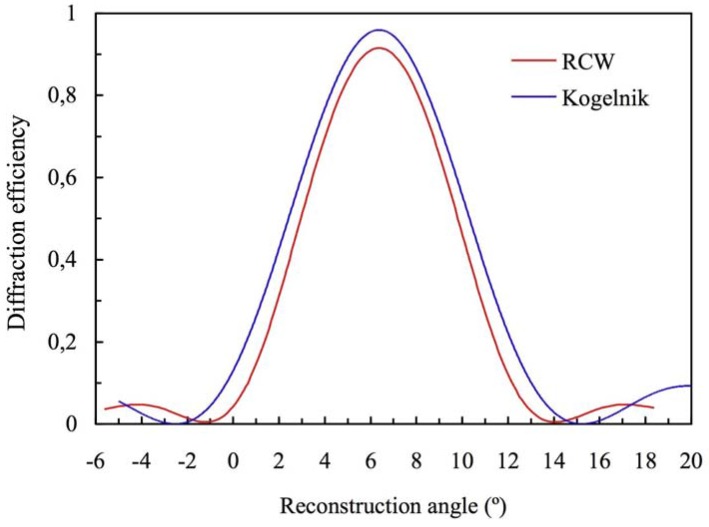
Diffraction efficiency of order +1 for volume transmission gratings with spatial frequency 350 lines/mm, *n*_1_ = 0.011 and 25 µm of thickness at first Bragg’s angle.

Although the cut-off spatial frequency in photopolymers avoid the high values of refractive index modulation at spatial frequencies of 350 lines/mm analyzed in the first section. It is interesting to analyze the discrepancies in the angular responses between Kogelnik’s theory and RCW in the most problematic case, gratings with the lowest spatial frequencies, 350 lines/mm, and high refractive index modulation, *n*_1_ = 0.011. In Figure 13 it can be seen the discrepancies in the results provided by these two theories. Therefore the approximations made in the Kogelnik’s analysis cannot be done in this case. When Kogelnik’s theory is used to fit the angular response of gratings with spatial frequency of 350 lines/mm important errors will be made in the values of thickness and refractive modulation.

## 4. Conclusions 

The limits of applicability of Kogelnik’s theory for gratings recorded in photopolymers have been studied. It has been demonstrated that for non-slanted transmission gratings recorded with spatial frequencies over 500 lines/mm, even for high refractive index modulations, Kogelnik’s theory is applicable in a range of thickness between 0 and 90 μm. For low values of the spatial frequency (under 500 lines/mm) the divergences between RCW method and Kogelnik’s theory appear when high values of refractive index modulation are considered. This is due to the existence of higher orders rather than the +1 and 0 ones. Nonetheless, the assumption that the second derivatives can be disregarded in the equations obtained from the coupled wave model can be considered valid for the range of refractive index modulations studied. This is evident if one compares the results of the CW and RCW theories, since for higher order the results obtained by using both theories agree. A comparison between theory and experimental work gives also evidence of the good behavior of Kogelnik’s theory to predict the efficiencies of orders 0 and +1. In this case non-slanted transmission diffraction gratings were recorded using a PVA/AA based photopolymer. For spatial frequencies over 1000 lines/mm there is good agreement between the experimental data of the angular responses of the efficiency of the first diffracted order with respect to the theoretical model proposed by Kogelnik.
